# Sense of agency in health and disease: A review of cue integration approaches^☆^

**DOI:** 10.1016/j.concog.2011.08.010

**Published:** 2012-03

**Authors:** J.W. Moore, P.C. Fletcher

**Affiliations:** a Institute of Cognitive Neuroscience, University College London, Queen Square, London, UK; b Department of Psychiatry, Herchel Smith Building for Mind and Brain Science, University of Cambridge, Addenbrooke’s Hospital, Cambridge CB2 2QQ, UK

**Keywords:** Volition, Voluntary action, Sense of agency, Cue integration, Bayesian, Schizophrenia, Neurological deficit

## Abstract

Sense of agency (SoA) is a compelling but fragile experience that is augmented or attenuated by internal signals and by external cues. A disruption in SoA may characterise individual symptoms of mental illness such as delusions of control. Indeed, it has been argued that generic SoA disturbances may lie at the heart of delusions and hallucinations that characterise schizophrenia. A clearer understanding of how sensorimotor, perceptual and environmental cues complement, or compete with, each other in engendering SoA may prove valuable in deepening our understanding the agency disruptions that characterise certain focal neurological disorders and mental illnesses. Here we examine the integration of SoA cues in health and illness, describing a simple framework of this integration based on Bayesian principles. We extend this to consider how alterations in cue integration may lead to aberrant experiences of agency.

## Highlights

► Established models of sense of agency (SoA) may be inadequate. ► We consider the relevance to SoA of a novel Bayesian cue integration framework. ► The value of this framework in understanding SoA in health and disease is discussed. ► Limitations of the model and avenues for future research are also discussed.

## 1 Introduction

Sense of agency (SoA) refers to the feeling of generating and controlling actions in order to influence events in the outside world. There have, traditionally, been two competing views of the cognitive origins of SoA. On the one hand it has been suggested that it arises principally from processes serving motor control (Blakemore, Wolpert, & Frith, 2002; Haggard, 2005). On the other, external, situational cues have been emphasised (Wegner, 2002, 2003).

In essence, these views differ according to whether they posit that SoA emerges from internal or external cues. More recently this dichotomy has been challenged by the suggestion that both sources of information are likely to be critical and that SoA depends on both internal and external cueing (Moore, Wegner, & Haggard, 2009; Synofzik, Vosgerau, & Newen, 2008a; Wegner & Sparrow, 2004; Wegner, Sparrow, & Winerman, 2004). In this paper we review evidence suggesting that multiple cues contribute to SoA and go onto introduce cue integration approaches that have proved successful in modelling human perception. In the context of this cue integration perspective, we consider psychiatric and neurological manifestations of disruptions in SoA.

## 2 Evidence for the contribution of multiple cues to SoA

Theories based on computational models of motor control suggest that SoA arises principally from internal cueing (from the very motoric signals responsible for generating the movement itself). Optimal motor control and learning require predictions of both the future states of the motor system and the sensory consequences of movement (Wolpert & Ghahramani, 2000). These predictions are derived from *internal forward models*, of which there are two classes: *forward dynamic* and *forward sensory*. The *forward dynamic model* captures the dynamics of bodily movement. The *forward sensory model* captures the causal relation between movements and their sensory consequences. The *forward model* is thought to play a key role in SoA (Blakemore et al., 2002; Frith, Blakemore, & Wolpert, 2000). According to the so-called ‘Comparator Model’ (CM) of SoA, predicted sensory information is compared with actual sensory information and, if there is a match, the sensory events are recognised as self-generated and SoA ensues. If there is mismatch, then the sensory information describes an external event, and SoA is attenuated or lost. This model has been used to explain a range of phenomena, such as the perceptual attenuation of self-generated stimuli (Blakemore, Wolpert, & Frith, 2000; Weiskrantz, Elliott, & Darlington, 1971) and the aberrant experience of agency characteristic of delusions of control (Blakemore et al., 2002; Frith, 2005).

In contrast, others have emphasised the role of external, situational cues, citing evidence that SoA can be modulated by priming participants with thoughts relevant to a movement just before it is made. For example, using this approach Wegner and Wheatley (1999) induced a false SoA for movements that participants had not in fact performed. Subsequent studies have replicated and extended this finding, showing that consistency between prime-induced prior thought and subsequent action can increase agency judgments for self-generated actions (Pronin, Wegner, McCarthy, & Rodriguez, 2006) and even enhances the experience of vicarious agency for another person’s actions (Wegner et al., 2004) and. As well as suggesting that SoA is fallible, these studies demonstrate that, even in the absence of internal motoric signals (when the participant plays no objective role in bringing about the outcome), SoA can occur (see Synofzik et al., 2008a, for extensive discussion of this).

Thus, SoA can be related both to internal motoric signals and to external cues. There is further compelling evidence from research based on the intentional binding (IB) paradigm. IB refers to the subjective binding in time of voluntary actions to their sensory consequences (Haggard, Clark, & Kalogeras, 2002). It has been proposed that IB provides a viable implicit measure of SoA (Moore & Haggard, 2010). Using this measure, Moore and Haggard (2008) demonstrated the contribution of both internal sensorimotor prediction and external action outcomes to SoA. Binding of the action to the outcome occurs when the likelihood of the outcome is strong, even in the absence of that outcome, i.e. sensorimotor prediction is sufficiently strong to produce binding. Furthermore, when sensorimotor prediction is weak, binding can occur but only when the outcome is present. On these trials the actual sensory outcome appears to trigger the binding effect retrospectively.

The dual contribution of internal motoric signals and external, situational cues was also demonstrated by Moore et al. (2009), again using the IB measure of SoA. As described above, priming the sensory consequences of movement can induce a false SoA (e.g. Wegner & Wheatley, 1999). By priming the sensory consequences of movement in the presence and absence of voluntary control over action, they investigated whether the presence of internal motoric signals modified the influence of external primes on SoA. It was found that both cues (primes and motoric signals) influence SoA. However, the influence of primes is significantly stronger when the action is *not* under the participants’ own voluntary control. This is intriguing, it suggests that such cues are not additive but rather interactive and that their relative influence is determined by their reliability: In both studies the external cues exert more influence over SoA when internal motoric signals are weak. To explain these findings Moore et al. (2009) proposed a form of optimal cue integration based on established models in human perception. In the following section we introduce these cue integration models of human perception, before considering their relevance to SoA.

## 3 Cue integration models in human perception

Accurate perception of features of the environment entails the use of multiple sources of information. Broadly speaking, there are two strategies for combining information: ‘sensory cue combination’, which maximises information from different sensory sources and ‘sensory cue integration’ which reduces the variance of the final perceptual estimate (Ernst & Bülthoff, 2004).

### 3.1 Sensory cue combination and integration

Because sensory information is inherently noisy, perception of an environmental feature based on a single sensory source would be unreliable. Our perceptual systems overcome this problem by generating percepts based on information derived from multiple sensory sources. When such information is complementary (i.e. not redundant), *sensory combination* is employed. This maximises the information provided by different sensory sources.

One benefit of this strategy is that it can disambiguate sensory information. For example, when sitting in a train carriage someone’s mobile phone may ring with the same tone as yours. The auditory information is insufficient for you to tell whether or not it is your phone ringing. Only by visually inspecting your phone (or using some other sensory information such as vibration), can you resolve this uncertainty. By combining different sensory sources you have increased the amount of relevant information available, leading to a more robust estimate.

Whilst sensory combination increases the amount of information from different sensory sources, sensory integration is a strategy which *reduces the variance* of the sensory estimate, thus boosting its reliability. Sensory integration occurs when sensory information from different sources is redundant; that is, the information refers to the same feature of the environment, and is in the same units and coordinates.

A fundamental goal for the perceptual system is to produce the most *reliable* estimate based on different sources of sensory information. By doing this an agent can effectively interact with its environment. ‘Maximum-Likelihood Estimation’ (MLE) is an optimal way of integrating different sources of sensory information, maximally reducing the variance of the final perceptual estimate. According to the MLE rule the final sensory estimate (S^) is the sum of individual sensory estimates (Eq. (1)) weighted by their reliability (the inverse variance of the sensory estimates; Eq. (2)). In this scheme, the variance of the final estimate is lower i.e. it is more reliable than estimates based on individual sensory sources.

(1) S^=∑iWiS^iwith∑iWi=1

(2) $$W_{i}=\frac{1/\sigma_{j}^{2}}{\sum_{i=1\ldots j\ldots N}1/\sigma_{j}^{2}}$$

Ernst and Banks (2002) have shown that, when combining visual and haptic information, humans estimate object size in a way that is entirely consistent with the use of MLE: when both types of cues are available, estimates are strongly influenced by (the more reliable) visual information, and when the experimenters degraded the quality of visual information (by adding noise), the influence of haptic information increased.

### 3.2 Incorporating prior information: Bayesian integration of cues

This MLE rule for sensory cue integration is entirely ‘bottom-up’; cue integration is modelled using likelihood functions only, thus ignoring the possible influence of prior information. However, such ‘top-down’ influences can be incorporated by adopting Bayes’ rule. Here, the cue weights are inversely proportional to the likelihood functions, and the integration of cues is influenced by prior information. Eq. (3) is the Bayesian scheme for estimating an object’s location (X) based on visual (V) and auditory cues (A; following Knill & Pouget, 2004).

(3) $$\overset{\text{Posterior}}{\overset{︷}{\text{P}(\text{X}|\text{V,A})}}=\overset{\text{Likelihood}}{\overset{︷}{\text{p}(\text{V,A}|\text{X})}}\overset{\text{Prior}}{\overset{︷}{\text{p}(\text{X})/}}\text{p}(\text{V,A})$$

Here, the posterior distribution is proportional to the product of the likelihood and the prior (the denominator is a normalising constant). The likelihood can be decomposed into the product of the likelihood functions associated with each of the cues (V, A) (Eq. (4)).

(4) p(V,A|X)=p(V|X)p(A|X)

What this means, in essence, is that the likelihood functions of each type of cue (visual and auditory) under a given set of circumstances, contribute to the overall “hypothesis” (posterior) about the location of the source of sensory stimulation. The posterior is a result of integration of the two cues dependent on their reliability. We have already shown how cue integration improves the estimate’s reliability. One advantage of the Bayesian scheme is that such reliability is further augmented: incorporating *prior information*, if it is available, further reduces estimation variance. A number of studies suggest that the human perceptual system can implement this form of Bayesian cue integration and it is this form of cue integration that, we suggest, underpins SoA (of course it should be noted that Eq. (4) entails an assumption about the absolute or conditional independence of V and A, and this will not necessarily hold for all situations or indeed for particular patient groups).

## 4 Cue integration and SoA

We suggest that the challenges facing the agency processing system are comparable to those facing the perceptual system. Accurate attribution of agency requires that uncertainty concerning the agentic origins of movement is dealt with appropriately. This uncertainty is inherent in the internal motoric signals underpinning sense of agency: sensorimotor prediction is probabilistic. Uncertainty is also a feature of the external cues to agency owing to the presence of noise in the sensory processing and transmission of these cues. Bayesian cue integration represents an optimal strategy for dealing with this uncertainty.

In this Bayesian cue integration framework, SoA can be viewed as the posterior (the belief or experience that one is the agent). The SoA will be determined by the integration of various agency cues, and the relative influence of these cues will be determined by their reliability. For example, consider a simplified case in which an agent has access only to sensorimotor (SM) and proprioceptive (PR) cues about the agentic origins of an action. In this example (Eq. (5)), the SoA (A) will be based on an integration of these two individual cue estimates of agency:

(5) $$\overset{\text{Posterior}}{\overset{︷}{\text{p}(\text{A}|\text{SM,PR})}}=\overset{\text{Likelihood}}{\overset{︷}{\text{p}(\text{SM,PR}|\text{A})}}\overset{\text{Prior}}{\overset{︷}{\text{p}(\text{A})/}}\text{p}(\text{SM,PR})$$

As in the example above, the likelihood would be the product of the likelihood functions associated with each of the cues (SM, PR) (Eq. (6)):

(6) p(SM,PR|A)=p(SM|A)p(PR|A)

This can be extended to incorporate other, external cues such as those provided in the study above (Moore et al., 2009). It provides a framework within which to understand how SoA depends on both internal and external cues with greater or lesser reliability. If one cue is absent, other cues can still be exploited allowing flexibility and robustness. By recognising the contribution of various agency cues we can also account for a wider range of findings than those models of SoA which emphasise the contribution of just one cue (see Section 4.1 below). Moreover, it acknowledges the interaction between cues: both of the IB studies described above found that the influence of external cues to agency increased when the reliability of internal motoric information decreased (Moore & Haggard, 2008; Moore et al., 2009). The relative resistance to external agency cues when internal motoric information is reliable suggests a higher intrinsic weighting for this latter source of information. Furthermore the increased influence of external agency cues when internal motoric information becomes less reliable suggests a shift in weighting in line with changes in reliability. This framework has also been invoked to explain the different pattern of results in patients with schizophrenia on SoA paradigms (Synofzik, Thier, Leube, Schlotterbeck, & Lindner, 2009; Voss et al., 2010), which we discuss in more detail below. It should be noted too that the prior information itself will have a certain degree of reliability and that strong, reliable priors may have a dominant influence on the ensuing SoA. We will consider this possibility – that expectations influence SoA profoundly.

As we have described, cue integration also results in the variance of the final estimate being less than the variance for each of the other cues individually. This improved reliability is important for SoA. In order to interact with, and exploit, its environment optimally, an agent has to determine those sensory events for which it is responsible. Such reliability is further augmented under the *Bayesian* approach to cue integration (compared with others such as the related Maximum Likelihood Estimate scheme): incorporating *prior information*, if it is known, reduces estimation variance. Recognising this offers a perspective on agency disruptions. We propose that SoA emerges from the integration of prior information and of internal and external cues. Furthermore, this integration is influenced by the reliability of each of these. According to this view, agency disruptions may be better understood by considering specific alterations in the nature and reliability of these signals of agency.

In short, the Bayesian approach acknowledges the importance of multiple cues in the emergence of SoA. The reliability of cues, as well as prior expectations, will determine the extent to which they contribute to this experience. Below, we consider the application of this view to relevant data in healthy adults before considering its utility in understanding disrupted SoA associated with certain disorders.

### 4.1 Further evidence in support of cue integration in SoA

In the domain of human perception, vision often has a higher intrinsic weighting than other perceptual modalities for certain estimates, such as size or location (Ernst & Banks, 2002; Ernst & Bülthoff, 2004). The data from Moore et al. (2009) suggest that, for SoA, internal cues to agency have a higher intrinsic weighting than external cues. Therefore, a strong prediction from the cue integration framework is that internal cues to agency will dominate SoA. As a consequence, the presence of such internal cues could result in illusory experiences of movement. Alternatively, the absence of internal cues to agency could result in a feeling of not having moved, when in fact one had. A recent study by Desmurget et al. (2009) supports these predictions. They directly stimulated parietal and premotor regions in seven patients undergoing brain surgery. Stimulation of inferior parietal regions (left and right) produced experiences of intending to move. Crucially, when stimulation was increased patients reported having actually moved despite no detectable evidence for this. This suggests that the internal cue to agency, the intention to move, was sufficient to produce the experience of movement, as is predicted by the cue integration model. Desmurget and colleagues also found that, when dorsal premotor regions were stimulated, patients did not experience an intention to move. Importantly, when stimulation intensity was increased this elicited detectable movements, but the patient reported no experience of moving. This suggests that the absence of the internal cue to agency, the intention to move, eliminated the experience of agency. Again, this is predicted by the cue integration framework. It should be noted that these patients could not see their own bodies. Visual cues, we would predict, should reduce SoA during parietal stimulation and increase it during premotor stimulation.

Importantly, these findings by Desmurget et al. are not readily explained by other models of SoA. For example, the CM (described in Section 1) states that SoA is dependent on the comparison between internal sensorimotor predictions and the sensory consequences of movement. However, in this study, patients experienced SoA in the absence of movement: no comparison was possible. An alternative model developed by Wegner and colleagues (Wegner, 2002, 2003; Wegner & Wheatley, 1999) also fails to explain this finding. According to this view SoA is based on a causal inference about the relationship between thought and action (and also action outcomes): for example, if a thought occurs prior to action, is consistent with the action and if there is no other possible cause of the action, then it is inferred that *I* caused by action. The patients who experienced SoA in the *absence* of movement are not consistent with the predictions of this model.

Subsequently presented external cues can also over-ride internal cues to agency such that the memory for agency may be profoundly changed. In the choice blindness (CB) paradigm, participants accept and then justify a choice they have not in fact made (Hall, Johansson, Tärning, Sikström, & Deutgen, 2010; Johansson, Hall, Sikström, & Olsson, 2005). One may invoke a cue integration explanation for this, whereby the external cue to agency (i.e. the actual presented external feedback about the choice) is sufficiently compelling to override any intrinsic signals about the choice that had been made. In other words the external cue receives higher weighting, and therefore contributes more to the experience, than internal cues. By this token, we would predict that, if the strength of the internal cue were augmented then participants would become more resistant to the experimenter’s subterfuge.

More recent IB data is also consistent with the cue integration framework. Desantis, Roussel, and Waszak (2011) investigated IB under different agency contexts: (a) participants were unsure whether they or a confederate had produced an outcome; (b) participants were told that they had produced the outcome; and (c) participants were told that the confederate had produced the outcome. They found normal binding in the uncertain condition, where there was no explicit instruction concerning the agentic origins of the outcome. They also found that IB was increased when participants were led to believe an outcome was contingent on their own behaviour, compared to the condition in which they were led to believe that the outcome was caused by the confederate. These results suggest that under uncertainty, participants use sensorimotor information to guide their experience of action, consistent with the idea that internal cues to agency receive higher weighting. However, if external cues to agency are sufficiently compelling (i.e. in the form of an instruction that the confederate is now in control), this may over-ride the sensorimotor information.

There is, then, convincing evidence that SoA can be characterised in terms of a weighted integration of internal and external cues. Moreover, this approach may explain a wider range of agency data than existing models. However, while we believe that the evidence presented in support of the cue integration approach is convincing, it remains, nevertheless, indirect. Perhaps more direct evidence could be obtained by adopting the approach used by Ernst and Banks (2002), independently manipulating the reliabilities of two agency cues.

In Moore, Wegner and Haggard’s (2009) paper it was suggested that cue integration may help explain disruptions in SoA associated with certain neurological disorders, an idea taken further in a commentary on that paper by Synofzik, Vosgerau, and Lindner (2009). In the following section, we expand on this idea. While evidence is limited, we would like to show that these disorders may be characterised in terms of changes in the how priors and cues are integrated, and that taking this perspective contributes to the goal of understanding how neurochemical and functional anatomical disturbances may manifest in an altered experience of agency.

## 5 Cue integration and disorders of SoA

Disturbed SoA may be present, sometimes strikingly so, both as a consequence of focal neurological lesions and of the, presumably more diffuse, neural changes underlying mental illness. We first consider certain well-known neurological conditions (Anarchic Hand Syndrome, Alien Hand Syndrome and Utilisation Behaviour) which are characterised by relatively well-demarcated disruptions before considering the more global deficits characteristic of schizophrenia.

### 5.1 Cue integration and neurological disorders

#### 5.1.1 Anarchic Hand Syndrome (AnHS)

AnHS (Marchetti & Della Sala, 1998) is characterised by the loss of sense of control for an affected limb. In contrast to Alien Hand Syndrome (see below) the sufferer recognises the hand as belonging to him or herself but there is a compelling experience that it is beyond control, having its own will and responding to external cues. In one reported instance (Feinberg, Schindler, Flanagan, & Haber, 1992), a patient remarked that his hand seemed to anticipate his own intentions, making a movement that he subsequently felt the desire to enact. It should be remarked that the movements of the hand are not utterly random: they appear goal-directed and sensitive to external stimuli. They may, moreover, be amenable to external verbal commands (Cantagallo, Spinazzola, Rabuffetti, & Della Sala, 2010). What is lacking is the sufferer’s sense of having willed the action or of being able to control or stop it. Nor is there some general deficit of agency: other movements are experienced as intended and voluntary (indeed, sometimes the non-affected hand is employed to physically control the anarchic movements of the affected hand).

#### 5.1.2 Utilisation Behaviour (UB)

In UB (Lhermitte, 1983), the patient’s actions are similarly driven by external stimuli. For example, he might don a pair of spectacles that happen to be lying in front of him. A second pair may then be picked up and worn on top of the first. In contrast to AnHS, UB is not experienced as counter-intentional or uncontrollable by the patient. Rather, it appears to be accepted without question. Indeed, the patient, when challenged, provides superficial explanations for it. As with AnHS, behaviour may be modified by verbal cues. For example, Lhermitte (1986) describes a patient who, on being told that he was in an art gallery, began to browse and comment on the pictures on the walls, a phenomenon that Lhermitte referred to as “Environmental Dependency Syndrome” (Lhermitte, 1986). It appears to be related, too, to imitative behaviour (*echopraxia*), perhaps another example of the behaviour being driven predominantly by external cues.

#### 5.1.3 Alien Hand Syndrome

While the term Alien Hand Syndrome (AlHS) is sometimes used interchangeably with AnHS, these two clinical syndromes are quite distinct. AlHS occurs in the context of a hemiparesis which the patient denies (anosognosia for hemiparesis). In addition to denying that the limb is paralysed, the patient behaves as though it is not, sometimes claiming that it moves normally and, in the case of AlHS, asserting that it does not belong to them (asomatognosia) and perhaps even belongs to someone else (somatoparaphrenia). Baier and Karnath (2008) reported an intimate link between a tendency to deny the existence of a hemiparesis and a disturbed sense of ownership for the affect limb, further identifying illusory body movements in a subset (5 out of 11) of individuals. Feinberg, Roane, and Ali (2000) showed a similar proportion of illusory movements and, further, reported that such illusions could be provoked by instructions to move the affected limb.

#### 5.1.4 AnHS, UB and AlHS: dissociable disturbances in cue integration?

There may be much to be gained by considering these syndromes together (e.g. Pacherie, 2007; Spence, 2002). While AnHS and UB are strikingly similar in a number of ways, most notably in the tendency towards behaviour which is driven by external cues rather than internal goals, they differ greatly in the accompanying subjective experience of the environmentally-driven behaviours.

Blakemore et al. (2002) have suggested that these two groups of patients differ in terms of their ability to represent (or access representations of) intended movements: AHS patients maintain this capacity whereas UB patients do not (presumably, these differences emerge because the underlying neurological deficits are distinct: AnHS is associated with lesions to supplementary motor area while UB is primarily associated with frontal damage (Lhermitte, 1983) though more widespread damage to fronto-striatal circuits may be critical (Archibald, Mateer, & Kerns, 2001)). With this in mind, we may speculate that both types of patient, on the surface at least, receive some external evidence that their behaviour is agentic. For example, they see and feel their hand moving and interacting with environmental cues. However, in the case of AnHS, there is a preserved capacity to recognise that external cues to agency are in direct conflict with their own goals, to which they have access. As such, patients will treat these external cues as unreliable indicators of agency. The consequence of this – preserved internal cues (indicating lack of agency) and unreliable external cues to agency – is the typical AnHS experience of a loss of SoA. In the case of UB, patients fail to recognise the fact that external cues to agency are in direct conflict with their own goals as they are unable to access these goal representations. In the absence of any perceived conflict, these external cues will be treated as reliable indicators of agency and patients will accept these movements as their own, justifying them with post hoc explanations. A comparable phenomenon in healthy people may be the choice blindness paradigm described above and the observations that external cues carry relatively more weight when internal cues are unreliable (Moore & Haggard, 2008; Moore et al., 2009).

While the AlHS, like AnHS, is characterised by a lack of SoA, the former also entails a denial of ownership. Synofzik and colleagues argue that considering AnHS and AlHS underline the importance of distinguishing ownership from agency (Synofzik, Vosgerau, & Newen, 2008b). This is a compelling argument: after all, how can we have SoA for a limb that we do not own? However, it is worth noting that, even when the sense of ownership is absent, the desire to move the affected limb can lead to the illusion or hallucination that it has indeed moved (Baier & Karnath, 2008; Feinberg et al., 2000). Moreover, this phenomenon shows a close relationship with both the degree of anosognosia (failure to recognise the hemiparesis) and disturbed sense of ownership. In our view, this relationship underlines the importance of the optimal integration of multiple cues in generating both the sense of ownership and of agency. Put simply, the sense of ownership emerges from somatosensory cues that are attenuated in such patients, with a corresponding attenuation of ownership experience. However, in the context of a conscious attempt to move the affected limb, the preserved intention may be sufficient to engender a (false) sense that movement has actually occurred. The internal motor cue that the movement is going to be made carries a comparatively greater weight than the noisy or absent sensory feedback and it is this that leads to the illusion that movement has occurred. This view leads to the prediction that the tendency to illusory movements under instruction would be greater in patients in whom anosognosia and asomatognosia is more pronounced, which is precisely what Feinberg and colleagues observed.

What is not clear is why AlHS is accompanied by the bizarre beliefs of the patient: the disavowal that the limb is paralysed or the assertion that it belongs to someone else. As Synofzik et al. observe, we may consider sense of ownership as emerging from multiple sensory cues and the absence of these cues in the paralysed limb might account for the feeling that it is an alien hand. But for the patient to arrive at the circumscribed delusional belief that it belongs to another is harder to fathom. In the next section, we consider cue integration in the context of the delusional state.

### 5.2 Cue integration and schizophrenia

A fundamental disturbance in SoA may plausibly be invoked to explain many of the positive symptoms (delusions and hallucinations) of schizophrenia. Passivity phenomena, quite clearly involve the misattribution of one’s own thoughts, feelings and actions to an external agent. In *thought insertion* for example, the deluded individual believes that his thoughts are not, in reality his own: they are actually those of another. In delusions of motor control, an action is perceived to have been initiated and controlled by another. In such cases, the misapplication of a SoA is obvious. But other features may also be characterised, albeit less obviously, by misattribution of agency. Auditory hallucinations clearly arise in the brain of the sufferer, perhaps as internal speech, but they are perceived as originating externally. There are a number of studies of reality distortion in schizophrenia that clearly show a bias towards attributing internally-generated percepts to an external source (Keefe, Arnold, Bayen, McEvoy, & Wilson, 2002; Simons, Davis, Gilbert, Frith, & Burgess, 2006). Of course, some of the other apparently irrational beliefs (delusions) of schizophrenia are less easy to explain in terms of agency misattribution. A delusion that one is being persecuted for example or the belief that general external events are referring specifically to oneself (delusions of reference) may be less amenable to this explanation. Yet these too have been considered in such terms (Frith, 1992), and it has been suggested that a fundamental deficit that leads to repeated confusion between internal and external origins of sense-data may be a prelude to the formation of such beliefs (e.g. Fletcher & Frith, 2009).

Thus, there are prima facie reasons for considering an altered SoA as being critical in explaining symptoms of schizophrenia. We propose that the Bayesian framework describing the emergence of SoA from integration of multiple cues may provide a powerful way of understanding this disruption more clearly and rooting it in the neurobiology of the disorder. We consider three possible disruptions: (1) an altered reliability of internal cues leading to a diminished recognition of internally generated movements; (2) a spurious enhancement of the salience and/or reliability of external cues leading to a greater tendency to attribute causal power to external rather than internal events; (3) a set of altered prior expectations leading to a greater tendency to make external attributions.

#### 5.2.1 Delusions and the reliability of internal cues

As we have suggested, internal cues may contribute in a variety of ways to SoA. For example, motor activity, sensory consequences and the extent to which the former predicts the latter, may be invoked to account for the sense that a particular action was internally generated. However, in schizophrenia, increased noise, perhaps arising from upregulated or chaotic dopamine firing, may obfuscate these normally reliable signals. It has been suggested that dopaminergic neuron firing codes the reliability of signals (Fiorillo, Tobler, & Schultz, 2003). Thus, a dysregulation in this coding might lead to a change in the signal-to-noise ratio and a corresponding attenuation of the degree to which these signals are able to contribute to the SoA. It would also lead to an increased likelihood of mismatch between the predicted and the actual sensory consequence of a movement, a mismatch which has been compellingly related to psychotic symptoms (Blakemore et al., 2002; Frith, 2005; Shergill, Samson, Bays, Frith, & Wolpert, 2005). Such a mismatch, referred to as prediction error, is a crucial learning signal (Schultz & Dickinson, 2000). As well as providing a useful framework for considering shifts in agency experience (as above), the idea of prediction error as a critical updating signal has been invoked to explain abnormal updating of beliefs about the environment in schizophrenia (see for example Corlett, Honey & Fletcher, 2007; Corlett, Frith, & Fletcher, 2009; Corlett, Honey, Krystal, & Fletcher, 2010; Fletcher & Frith, 2009).

Recent evidence strongly supports the idea that, in patients with schizophrenia, these internal signals are corrupted by noise. Voss and colleagues (2010) used the same probabilistic IB paradigm developed by Moore and Haggard (2008; see Section 1). People with Schizophrenia showed a strong predictive contribution to IB in *both* the 50% and the 75% conditions. In healthy controls the predictive contribution was much stronger in the 75% condition (replicating Moore & Haggard’s, 2008, finding). These data show that prediction in schizophrenia was noisy: The predictive contribution to IB was insensitive to actual outcome probability. Intriguingly, people with schizophrenia also showed an increase in the contribution of external agency cues to IB. This is predicted by the cue integration framework: The SoA is dominated by the most reliable source of information.

This finding is compatible with a study by Synofzik, Thier, Leube, Schlotterbeck, and Lindner (2010) using an agency attribution paradigm. They showed that agency judgments in people with schizophrenia relied more strongly on visual feedback about an action rather than on internal sensorimotor cues. Moreover, the degree of reliance on external visual feedback appeared to be associated with the reliability of internal sensorimotor information about the action: The variability of internal sensorimotor information in patients was approximately twice that of controls, and the influence of external visual feedback about the action in patients was about twice that of controls. This is compatible with predictions made within a cue integration framework.

One might further predict that a reduction in the reliability of internal signals could lead to a weakening of the sense of bodily ownership, a suggestion in keeping with the increased propensity of people with schizophrenia to experience illusory body ownership (Peled, Ritsner, Hirschmann, Geva, & Modai, 2000). In short, noisy (and therefore unpredictable) internal signals produce an enhanced tendency to attribute changes to external agents rather than to oneself.

#### 5.2.2 Delusions and the spurious enhancement of external cues

Previous models of schizophrenia have suggested that environmental stimuli and events acquire an importance or salience that leads to their incorporation into the sufferer’s beliefs about of the world. This model of excessive salience has been linked to dopaminergic dysregulation and has been invoked to account for both the symptoms and the characteristic response to treatment (Kapur, 2003). Moreover, frequent reference has been made to the observation that patients with schizophrenia often see meaningful links between fortuitously co-occurring events (so-called “apophenia”). Schneider (1930) commented on a pronounced tendency to see connections both between coincident external events and between simultaneously occurring external event and internal “impressions” or percepts.

This alteration in the perceived salience of external events, and the corresponding reallocation of attention towards them (and away from internal signals), may alter the weightings applied in the process of cue integration. This could lead to a fundamental alteration in the SoA. Spurious but compelling associations between external occurrences, or between external events and internal experiences, could lead to a failure to attribute correctly an event to oneself. For example, if, as one moved, there was a coincident noise (a car horn perhaps), then the enhanced, inappropriate association between one’s movement and the noise might lead to the experience that one had caused the noise by one’s movement, or, alternatively, that the sensation of movement was in some way caused by the noise. The latter could ultimately lead to the delusion of control, particularly when compounded by the noisy sensorimotor signals discussed above.

#### 5.2.3 Delusions and prior expectations

There are many examples in healthy people of the illusion of being in control when one is not (e.g. Langer, 1975) or, conversely, of the sense that one’s own movements are actually under some external control. Such illusions of control can arise as a consequence of sufficiently strong prior beliefs. A good example of this is the “table-turning” phenomenon wherein groups of necromancers experience a table moving, apparently under the control of the spirit world. Using force sensors, Michael Faraday (1853) showed that actually, the movements of the table depended upon forces from the living rather than the dead. Ouija boards, hypnotism and facilitated communication are further examples of how prior beliefs fundamentally alter the experience of SoA or, in Wegner’s elegant description: ‘…the simple belief that the action can come from the other person is the main basis of action projection. It is the attribution to outside agency, in other words, that helps to fuel the loss of conscious will. Once the belief in outside agency is in place, the processes for interpreting one’s own action are rocked at the base’ (Wegner, 2002, p. 199).

This is a phenomenon that has been replicated experimentally (Wegner, Fuller, & Sparrow, 2003). Prior expectations clearly, therefore, influence on SoA. This is important to bear in mind when considering delusions. Recent models of psychotic experience and belief have considered the importance of altered or aberrant priors (see Corlett et al., 2009, 2010; Fletcher & Frith, 2009). Such ideas are necessarily speculative but the clear tendency of people with delusions to use prior information in distinct, sub-optimal ways (e.g. Woodward, Buchy, Moritz, & Liotti, 2007; Woodward, Moritz, & Chen, 2006) must be considered relevant to their experiences of action and agency particularly when so many of their symptoms involve erroneous external attribution. In short, once the belief has formed that one is under external control, particularly in the setting of noisy internal cueing, this belief generally shapes the interpretation of one’s experiences.

## 6 Concluding remarks

We have reviewed and elaborated on the cue integration approach introduced by Moore et al. (2009), placing this approach within the context of well-established cue integration models of human perception and outlining its possible contribution to the understanding of SoA in both health and disease. We acknowledge that much of this is speculative though argue that there are a number of instances, in the context of both normal and disrupted SoA, where this perspective – that weighted integration of multiple, internal and external, cues together with prior beliefs contributes to and shapes the emergent SoA – provides a useful framework for understanding experimental and clinical phenomena. Clearly further research explicitly testing the predictions made by the cue integration framework is required. One approach, as we suggest above, would be to adopt paradigms used in previous research on human perception (e.g. Ernst & Banks, 2002) in which the reliability of several cues is systematically manipulated and dissociated.

Other outstanding issues concern the specific details of the framework. For example, the contribution of internal motoric cues to SoA is likely to be asynchronous with external agency cues based on sensory feedback about the action, which are necessarily processed later. Such asynchrony can be problematic for cue integration (Ernst & Bülthoff, 2004). One possible solution would be to consider a dynamical cue integration mechanism that updates its estimates as new information becomes available (these are known as ‘Kalman filter’ approaches).

A recurring theoretical problem in the discussion of Bayesian frameworks such as this concerns the origins of ‘priors’. One possibility is that there may be a natural tendency to be biased towards ascribing agency to oneself under conditions of uncertainty (e.g. Desantis et al., 2011; Farrer et al., 2008), i.e. the prior for “self” is inherently stronger than for “other”. We can only speculate on whether or not this might confer some advantage. However, it appears that, even in infants, there is a remarkable ability to flexibly shift between self- and other-attributions (see Gweon & Schulz, 2011). This suggests that, if there is an inherently greater prior for self under uncertainty, it is easily overcome by experience. One thing that is worth noting is that if, as we suggest happens in schizophrenia, one repeatedly but unsuccessfully tries to minimise error and uncertainty, then this could enhance the prior for external rather than internal control. This in turn may lead to the strong belief that one is being controlled and persecuted by some external agent, with this belief itself acting as a strong prior in further interactions with the world. Key to this proposal is ‘abductive inference’, or inference to the best explanation. Abductive inference was raised and thoroughly examined by Charles Pierce at the turn of the 19th century (see (Peirce, Putnam, & Ketner, 1992)). It involves determining the most likely cause of an *unexpected* event. As we have described above, persistent error signalling is implicated in the pathogenesis of schizophrenia. Over time the patient will repeatedly experience the sensory consequences of their actions as being *unexpected*. The patient may conclude, therefore, that they are not in control of their actions (see Coltheart, Menzies, & Sutton, 2010, for a review of the putative role of abductive inference in delusion formation). As we suggest above, once the belief has formed that one is under external control, particularly in the setting of noisy internal cueing, the interpretation of one’s experiences is shaped by this belief.

Despite the current limitations, which arise from a lack of evidence more than from a failure of the explanatory power of the framework, we suggest that the cue integration approach holds real promise in shedding light on the cognitive processes supporting SoA. In particular, its application to schizophrenia may prove important in relating the subjective experiences that characterise the disorder to the underlying neural and neurochemical disturbances.
